# Vanadium Derivative Exposure Promotes Functional Alterations of VSMCs and Consequent Atherosclerosis via ROS/p38/NF-κB-Mediated IL-6 Production

**DOI:** 10.3390/ijms20246115

**Published:** 2019-12-04

**Authors:** Chang-Ching Yeh, Jing-Yiing Wu, Guan-Lin Lee, Hsiu-Ting Wen, Pinpin Lin, Cheng-Chin Kuo

**Affiliations:** 1 Cellular and System Medicine, National Health Research Institutes, Zhunan 35053, Taiwan; ccyyehcc@nhri.org.tw (C.-C.Y.); jywu@nhri.org.tw (J.-Y.W.); lgl0311@gmail.com (G.-L.L.); 060334@nhri.org.tw (H.-T.W.); 2 Graduate Institutes of Life Sciences, National Defense Medical Center, Taipei 114, Taiwan; 3 National Institute of Environmental Health Sciences, National Health Research Institutes, Zhunan 35053, Taiwan; pplin@nhri.org.tw; 4 Graduate Institute of Basic Medical Science, China Medical University Hospital Taichung, Taichung 404, Taiwan

**Keywords:** vanadium derivatives, ROS generation, IL-6 production, VSMCs, atherosclerosis

## Abstract

Vanadium is a transition metal widely distributed in the Earth’s crust, and is a major contaminant in fossil fuels. Its pathological effect and regulation in atherosclerosis remain unclear. We found that intranasal administration of the vanadium derivative NaVO_3_ significantly increased plasma and urinary vanadium levels and induced arterial lipid accumulation and atherosclerotic lesions in apolipoprotein E-deficient knockout mice (*ApoE^−/−^*) murine aorta compared to those in vehicle-exposed mice. This was accompanied by an increase in plasma reactive oxygen species (ROS) and interleukin 6 (IL-6) levels and a decrease in the vascular smooth muscle cell (VSMC) differentiation marker protein SM22α in the atherosclerotic lesions. Furthermore, exposure to NaVO_3_ or VOSO_4_ induced cytosolic ROS generation and IL-6 production in VSMCs and promoted VSMC synthetic differentiation, migration, and proliferation. The anti-oxidant *N*-acetylcysteine (NAC) not only suppresses IL-6 production and VSMC pathological responses including migration and proliferation but also prevents atherosclerosis in *ApoE^−/−^* mice. Inhibition experiments with NAC and pharmacological inhibitors demonstrated that NaVO_3_-induced IL-6 production is signaled by ROS-triggered p38-mediated NF-κB-dependent pathways. Neutralizing anti-IL-6 antibodies impaired NaVO_3_-mediated VSMC migration and proliferation. We concluded that NaVO_3_ exposure activates the ROS-triggering p38 signaling to selectively induce NF-κB-mediated IL-6 production. These signaling pathways induce VSMC synthetic differentiation, migration, and proliferation, leading to lipid accumulation and atherosclerosis.

## 1. Introduction

Increasing evidence has indicated that exposure to environmental pollutants such as particulate matter (PM) and ultra-fine particles (UFPs), particularly residual oil fly ash (ROFA), lead to systemic pro-oxidant accumulation and inflammation, both of which play a critical role in the development of cardiopulmonary diseases [1,2], a major health issue worldwide with significant morbidity and mortality [3]. The main underlying cause of cardiovascular diseases is atherosclerosis, which is recognized as a chronic inflammatory disease [4]. Evidence has indicated that exposure to PM air pollution elicits numerous pathological and biological responses, including systemic inflammation and oxidants, which increases the relative risk of cardiovascular mortality [2,5,6,7]. Although the toxic effects of PM on cardiovascular disease are usually assessed by its particle mass, there is increasing evidence that the chemical composition and size/morphology of PM may be a major contributor to its pathotoxicity toward the cardiovascular system [6,8]. Vanadium is one of the metal compounds found in atmospheric suspended matter and is classified as PM2.5 by its size. Considering the rich speciation chemistry of vanadium, the specific forms of vanadium oxides are likely a combination V_2_O_5_, VO_2_, V_2_O_3_, and some of their salts [9]. Suspended particles containing vanadium are inhaled into the respiratory system and reach the circulation and organs, adversely affecting the individual’s physical health [10,11]. Inhalation of air containing vanadium can lead to pathological effects on human organ systems including the respiratory system, lungs, neurological system, eyes, hematopoietic tissue, immune system, and cardiac physiology, potentially through the induction of reactive oxygen species (ROS) and inflammatory responses [10,11,12]. These inhalations studies with mice have demonstrated that the form of vanadium is critical, with some being much more toxic than others [13,14].

Metallic vanadium, due to several oxidation states, forms numerous inorganic compounds including vanadyl sulfate, sodium metavanadate, sodium orthovanadate, and vanadium pentoxide. The pentavalent form (VO^3−^) and quadrivalent form (VO^2+^) of vanadium exist in extracellular and intracellular body fluids, respectively, which are found to regulate the activity of various metabolic enzymes. Therefore, a low concentration (μg) of vanadium is involved in the development and growth of organisms and is considered essential for maintaining health. Furthermore, vanadium derivatives have been used as a common supplement to enhance athletes’ weight training at doses up to 60 mg/d [15]. In vitro and in vivo studies have shown that vanadium compounds have anti-diabetic effects by increasing glucose transport activity and improving glucose metabolism [16]. Recently, vanadium compounds are being considered for the application in cancer treatment [17]. However, high air concentrations of vanadium occur in the occupational setting as a result of the presence of vanadium oxides in dust, leading to humans being exposed to an excessive amount of vanadium, with resulting toxicity. There is growing evidence that inhalation of vanadium-rich environmental pollutions, particularly vanadium pentoxide, can cause damage to human organ systems [10,11,12]. In addition, vanadium pentoxide is classified as possibly carcinogenic to humans by the IARC (International Agency for Research on Cancer). These observations indicate that vanadium derivatives with different physical and chemical properties exhibit different physicochemical properties, which confer beneficial properties or toxic effects. However, their adverse effects and susceptibility to vascular diseases such as atherosclerosis and the pathological mechanisms involved remain undetermined. 

It is well-known that mice are most frequently used in various diseases such as atherosclerosis, a multifaceted disease which can involve a pathological progression. Therefore, the choice of the right transgenic mouse model is vital for atherosclerosis research. Since wild-type mice are not thought to develop atherosclerosis unless challenged for long periods with Western diets, transgenic mouse models of atherosclerosis have been developed to study experimental atherosclerosis. The first line of apolipoprotein E-deficient knockout mice (*ApoE^−/−^*) was developed in 1992. Further, *ApoE^−/−^* mice are frequently used and are valuable tools in atherosclerosis research. Notably, *ApoE^−/−^* mice can develop extensive atherosclerotic lesions on a chow diet, and the resulting atherosclerotic lesions are comparable to human lesions. Therefore, *ApoE^−/−^* mice have been used to develop new drugs against atherosclerosis. Accordingly, *ApoE^−/−^* mice, but not wild-type mice, were used in this study to investigate the effect of vanadium derivatives, including NaVO_3_ and VOSO_4_, on atherosclerosis. 

Excessive and inappropriate activation of the innate immune system has been implicated in the development of chronic metabolic diseases including atherosclerosis and systemic inflammation [4,18,19,20]. In general, the mechanisms underlying the induction of inflammatory discords leading to most pathological conditions remain to be determined. However, a disturbance in the reduction–oxidation (redox) equilibrium of cells and tissues may lead to an overwhelming proinflammatory state, which leads to cellular dysfunction and tissue injury. ROS such as superoxide, hydrogen peroxide, and hydroxyl radicals are highly reactive and thus harmful to health, causing chronic diseases such as atherosclerosis [21,22], a complex chronic vascular disease that progressively occludes the lumen of large and medium-sized arteries with plaques. This initiates with endothelial injury followed by immune and proinflammatory cell accumulation, lipid deposition, and progressive inflammatory responses [4,23,24]. Further, the inflammatory responses in the microenvironment of atherosclerotic lesions drive medial smooth muscle cell migration and proliferation into the intima, consequently causing plaque formation, which is a key event in the pathophysiology of atherosclerosis [3,25,26,27]. Vascular smooth muscle cells (VSMCs) reside in the media of normal blood vessels, where they are quiescent and assume a contractile phenotype. Under pathological conditions, VSMCs transform into a highly proliferative synthetic phenotype with a loss of contractile markers (SM22α, α-SM actin, and E-cadherin) and induction of synthetic markers such as vimentin [3,27]. Furthermore, they contribute to vascular inflammation by producing proinflammatory cytokines such as interleukin 6 (IL-6) [28]. These VSMC pathophysiological alterations cause atherosclerotic lesions and plaque formation. 

Given that ROS and inflammation play a pathogenic role in atherosclerosis and vanadium exposure can promote cellular ROS and inflammation, we hypothesized that excessive vanadium exposure may have pathological significance in VSMC survival, proliferation, and/or migration as well as atherosclerosis. Our results from in vivo and in vitro analyses reveal that vanadium derivatives, VOSO_4_ and NaVO_3_, selectively induces IL-6-dependent VSMC pathological responses including phenotypic alternations, migration, and proliferation, with consequent atherosclerotic plaque formation which is mediated by NADPH oxidase-derived ROS generation, leading to p38-mediated NF-κB (nuclear factor kappa light chain enhancer of activated B cells) activation and NF-κB-dependent IL-6 production.

## 2. Results

### 2.1. Intranasal Administration of NaVO_3_ Induces Atherosclerosis in ApoE^−/−^ Mice

**Figure 1:**
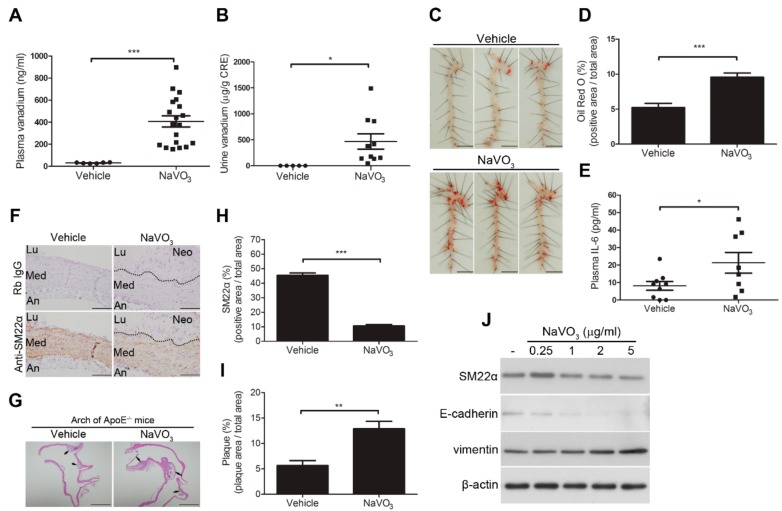
Intranasal administration of NaVO3 induces atherosclerosis in apolipoprotein E-deficient knockout mice (*ApoE^−/−^*) mice. *ApoE^−/−^* mice were intranasally administered NaVO3 (4 mg/kg) once a week for 12 weeks. (**A**) Plasma vanadium levels in vehicle (endotoxin-free water)-treated mice (*n* = 6) and NaVO3-treated mice (*n* = 19) and (**B**) urine vanadium levels in mice treated with vehicle (*n* = 5) and NaVO3 (*n* = 10) were measured by ICP (Inductively coupled plasma)–mass spectrometry. The levels of vanadium in urine were normalized by creatinine (CRE). Each dot denotes an individual mouse. The solid black line denotes the mean value. (**C**) Lipid contents in aorta from mice treated with vehicle and NaVO3 were analyzed by Oil Red O staining. Scale bars represent 5 mm. (**D**) The area of positive staining for Oil Red O was quantified using ImageJ software as a percentage of total aortic area (*n* = 9 vehicle; *n* = 8 NaVO3). (**E**) Plasma interleukin 6 (IL-6) levels in vehicle treatment (*n* = 9) and NaVO3 treatment (*n* = 8) were measured by ELISA. (**F**) SM22α content in aorta tissues from mice was analyzed by immunohistochemistry. Immunopositive areas shown are shown at 400× magnification. An: adventitia; Lu: lumen; Neo: neointima; Med: media. Negative control represents staining with an irrelevant isotype control antibody. Scale bars represent 100 µm. (**G**) Paraffin-embedded tissues with atherosclerotic plaque (black arrow) were observed by hematoxylin and eosin (H&E) stain at 40× magnification. Scale bars represent 1 mm. (**H**) Immunopositive areas and atherosclerotic plaque in paraffin-embedded aorta tissues of mice treated with vehicle (*n* = 5) or NaVO3 (*n* = 10) were quantified using ImageJ software as a percentage of total aortic area in each section. Data in (**A**,**B**), (**D**,**E**), and (**H**,**I**) represent mean ± SEM. (**J**) Vascular smooth muscle cells (VSMCs) were treated with a different amount of NaVO3 for 48 h. Cell lysates were immunoblotted with antibodies for SM22α, E-cadherin, vimentin, or β-actin. Experiments were repeated three times with similar results. Data represent mean ± SEM of three experiments. * *p* < 0.05; ** *p* < 0.01; *** *p* < 0.001.

This study showed that the plasma and urinary vanadium concentrations significantly increased in NaVO_3_-exposed mice (mean 407.5 ± 50.4 ng/mL in plasma, (*n* = 19) and 469 ± 147.4 µg/g creatinine in urine, (*n* = 10)) as compared to control mice exposed to endotoxin-free water (mean 30.41 ± 1.881 ng/mL in plasma, (*n* = 6) and 0.69 ± 0.18 µg/g creatinine in urine, (*n* = 5)) (Figure 1A,B), suggesting that intranasal administered NaVO_3_ can be absorbed into the circulation system. Furthermore, intranasal administration of NaVO_3_ induced arterial lipid accumulation in the murine aorta but did not affect circulating lipid levels (cholesterol and triglyceride) compared to vehicle-exposed mice (Figure 1C,D and Figure S1), and was accompanied by increasing plasma IL-6 levels (Figure 1E). Notably, there were no damaging effects to the kidney, liver, or heart, but mild lung inflammation including inflammatory leukocyte infiltration was observed in the NaVO_3_-exposed mice (Figure S2), suggesting that intranasal administration of NaVO_3_ has a major effect on the arteries rather than the lungs and other organs. These results suggest that NaVO_3_ may be an atherosclerosis initiator or inducer.

The decrease in VSMC differentiation marker proteins such as SM22α in atherosclerotic lesions is a common characteristic of atherosclerosis [3,29], we determined whether intranasal administration of NaVO_3_ significantly decreased SM22α. A significant decrease in the immunopositive areas for SM22α in the atherosclerotic lesions (Figure 1F) was found to be highly associated with enhanced neointimal formation in the atherosclerotic lesion of NaVO_3_-exposed mice (Figure 1G). Quantitative analysis confirmed significant downregulation of SM22α in the atherosclerotic plaque of NaVO_3_-exposed mice as compared to vehicle-treated mice (Figure 1H–I). In addition, NaVO_3_ significantly modulated the expression of VSMC differentiation protein markers, decreased SM22α and E-cadherin, and increased vimentin in a dose-dependent manner (Figure 1J). These results suggest that NaVO_3_ induces atherosclerotic lesions by suppressing SM22α and E-cadherin and increasing vimentin expression to alter VSMC phenotype switching.

### 2.2. VOSO_4_ and NaVO_3_ Promote Pathophysiology of VSMC In Vitro

**Figure 2:**
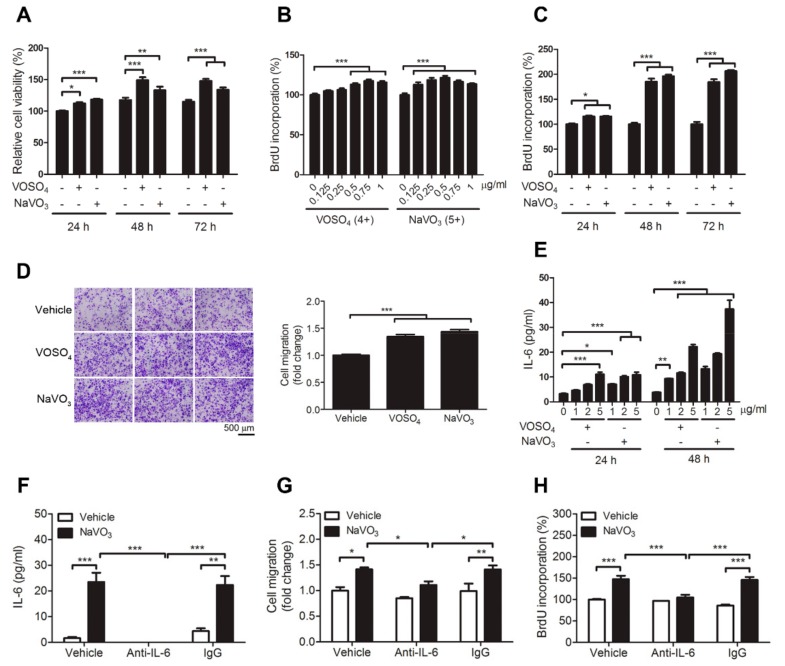
VOSO_4_- and NaVO_3_-induced VSMC migration and proliferation require interleukin 6 (IL-6) production. (**A**) VSMCs were incubated in 0.5% fetal bovine serum (FBS) in DMEM with VOSO_4_ (1 µg/mL) or NaVO_3_ (1 µg/mL) for 24, 48, and 72 h. MTT assay was used to determine cell viability. (**B**) VSMCs were incubated in 0.5% fetal bovine serum (FBS) in DMEM with different amounts of VOSO_4_ or NaVO_3_ for 24 h. (**C**) Quiescent VSMCs were treated with VOSO_4_ (1 µg/mL) or NaVO_3_ (1 µg/mL) for 24, 48, and 72 h. BrdU incorporation assay was used to determine cell proliferation. (**D**) Quiescent VSMCs stimulated with vehicle (endotoxin-free water), VOSO_4_ (1 µg/mL), or NaVO_3_ (1 µg/mL) for 24 h. VSMC migration was measured by the transwell assay with PDGF (platelet-derived growth factor)-BB as a chemoattractant. Scale bar represents 500 μm. (**E**) VSMCs were treated with different amounts of VOSO_4_ or NaVO_3_ for 6, 24, and 48 h. IL-6 levels in culture supernatants were measured by ELISA. (**F**–**H**) VSMCs were treated with vehicle or NaVO_3_ (1 µg/mL) with or without anti-IL-6 (2.5 μg/mL) or control IgG (2.5 µg/mL) for 24 h. (**F**) IL-6 levels in culture supernatants were measured by ELISA. (**G**) VSMC migration was then measured by the transwell assays. (**H**) VSMC proliferation was measured by BrdU incorporation assay. Data represent mean ± SEM of three experiments. * *p* < 0.05; ** *p* < 0.01; *** *p* < 0.001.

MTT assays showed that VOSO_4_ and NaVO_3_ increased VSMC cell viability (Figure 2A). Consistent with MTT assays, BrdU (Bromodeoxyuridine / 5-bromo-2′-deoxyuridine) incorporation in cells treated with VOSO_4_ and NaVO_3_ was increased in a dose-dependent and time-dependent manner when compared to those treated with vehicle (Figure 2B,C). We next used a transwell migration assay to determine whether VOSO_4_ and NaVO_3_ affected VSMC migration. The results revealed that VOSO_4_ and NaVO_3_ significantly increased VSMC migration (Figure 2D) when compared with vehicle control. These results indicate that vanadium salts, NaVO_3_, and VOSO_4_, can promote VSMC proliferation, differentiation, and migration.

### 2.3. IL-6 Is Essential for VOSO_4_- and NaVO_3_-Induced VSMC Proliferation and Migration

Given that NaVO_3_ induced atherosclerotic lesions and VSMC phenotype alternation accompanied by increasing plasma IL-6 levels (Figure 1), we wondered if IL-6 secreted by VSMCs may be involved in vanadium salt-induced VSMC migration and proliferation. Our results revealed that VOSO_4_ and NaVO_3_ induced IL-6 production in VSMCs in a time-dependent and dose-dependent manner (Figure 2E). Furthermore, IL-6 neutralizing antibodies but not control IgG significantly inhibited not only NaVO_3_-induced IL-6 production but also NaVO_3_-induced migration and proliferation of VSMCs (Figure 2F–H), suggesting that NaVO_3_-induced VSMC proliferation and migration is mediated by the release of IL-6 into the extracellular milieu.

### 2.4. ROS-Mediated IL-6 Is Essential for NaVO_3_-Mediated VSMC Functions

**Figure 3:**
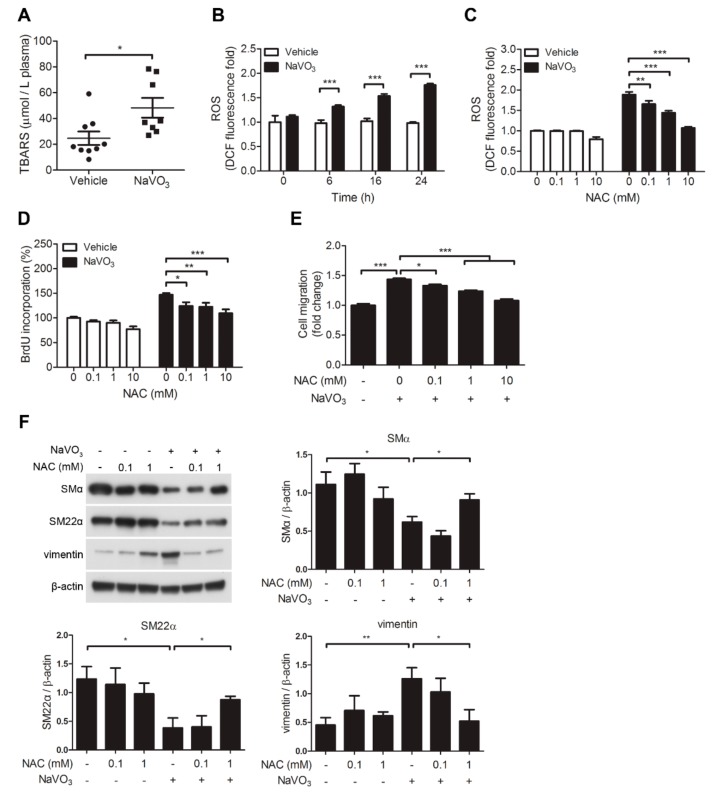
Reactive oxygen species (ROS)-mediated IL-6 is essential for NaVO_3_-mediated VSMC migration and proliferation. (**A**) Plasma ROS levels in mice treated with vehicle (endotoxin-free water) (*n* = 9) or NaVO_3_ (*n* = 8) once weekly for 12 weeks were measured by thiobarbituric acid reactive substances (TBARS) assay. The solid black line denotes the mean value. (**B**) Quiescent VSMCs were treated with NaVO_3_ for the indicated time. Intracellular ROS levels were measured by 6-carboxy-2,7-dichlorodihydrofluorescein diacetate (DCFDA). (**C**–**E**) After pretreating VSMCs with different concentrations of *n*-acetyl-l-cysteine (NAC) for 30 min, cells were stimulated with vehicle or NaVO_3_ (1 µg/mL) for 24 h. (**C**) Intracellular ROS levels and mitochondrial ROS were measured by DCFDA. (**D**) VSMC proliferation was measured by BrdU incorporation assay. (**E**) VSMC migration was then measured by the transwell assays. (**F**) VSMCs were treated with NaVO_3_ (1 µg/mL) with or without different concentrations of NAC for 48 h. Cell lysates were immunoblotted with antibodies for vimentin, smooth muscle α-actin (SMα), SM22α, or β-actin. Densitometry analysis of SMα, SM22α, and vimentin protein expression relative to β-actin. Data represent mean ± SEM of three experiments. * *p* < 0.05; ** *p* < 0.01; *** *p* < 0.001.

Our results revealed that plasma ROS levels were increased in NaVO_3_-exposed mice with atherosclerosis compared with vehicle control mice (Figure 3A). In addition, we wondered if NaVO_3_ also induced VSMC ROS generation. Intracellular ROS level as assessed by the cell-permeable dye 6-carboxy-2,7-dichlorodihydrofluorescein diacetate (DCFDA) was increased in VSMCs exposed to NaVO_3_ in a time-dependent manner (Figure 3B). Notably, this ROS induction was suppressed by *N*-acetylcysteine (NAC) in a dose-dependent manner (Figure 3C), which was accompanied by blocking VSMC proliferation and migration without affecting VSMC viability (Figure 3D–E and Figure S3A). In addition, NAC dose-dependently recused NaVO_3_-mediated reduction of SMα and SM22α and enhancement of vimentin (Figure 3F). These results suggest that cytosolic ROS participates in NaVO_3_-mediated VSMC pathology.

**Figure 4:**
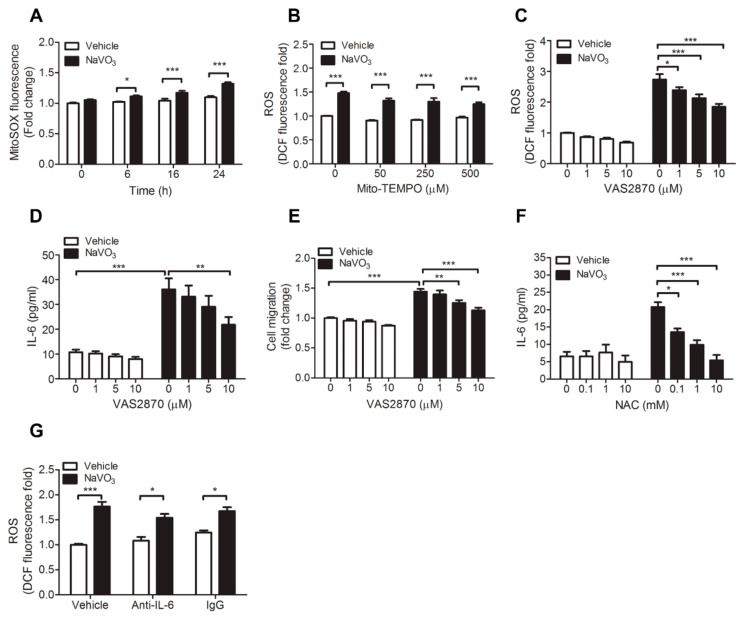
NADPH oxidases-derived ROS contributes to NaVO_3_-induced VSMC pathological responses. (**A**) VSMCs were treated with endotoxin-free water (vehicle) or NaVO_3_ (1 µg/mL) for the indicated times. Mitochondrial ROS were measured by MitoSOX. (**B**) VSMCs were pretreated with different concentrations of mito-TEMPO for 30 min, then stimulated with vehicle or NaVO_3_ for 24 h and intracellular ROS levels were measured by DCFDA. (**C**–**E**) After pretreating VSMCs with different concentrations of NADPH oxidase inhibitor VAS2870 for 30 min, cells were stimulated with vehicle or NaVO_3_ for 24 h. (**C**) Intracellular ROS levels were measured by DCFDA. (**D**) IL-6 levels in culture supernatants were measured by ELISA. (**E**) VSMC migration was then measured by the transwell assays. (**F**) IL-6 levels in culture supernatants of VSMCs treated with vehicle or NaVO_3_ with or without different concentrations of NAC for 24 h were measured by ELISA. (**G**) ROS levels in VSMCs treated with vehicle or NaVO_3_ (1 µg/mL) with anti-IL-6 (2.5 μg/mL) or control IgG (2.5 µg/mL) for 24 h were measured by DCFDA. Data represent mean ± SEM of three experiments. *p < 0.05; **p < 0.01; ***p < 0.001.

Because mitochondrial-derived ROS is an important source of cytosolic ROS, we thus investigated whether mitochondrial-derived ROS also contributes to NaVO_3_-induced intracellular ROS production. MitoSOX Red fluorescence assay, which exclusively measures ROS in the cytosol, showed that mitochondrial ROS level only slightly increased in NaVO_3_-treated VSMCs (Figure 4A). Furthermore, mito-TEMPO, mitochondria-specific superoxide scavenger, did not affect NaVO_3_-induced ROS generation (Figure 4B), suggesting that mitochondria are not major sources of cytosolic ROS.

In addition to mitochondria, the NADPH oxidases family is thought to be responsible for the production of cytosolic ROS [30,31]. We thus used NADPH oxidases pharmacological inhibitor VAS2870 to investigate whether NADPH oxidases involve in NaVO_3_-induced cytosolic ROS production. The results revealed that VAS2870 dose-dependently reduced NaVO_3_-induced cytosolic ROS production in VSMCs and had no effect on VSMC viability. (Figure 4C and Figure S3B). This is accomplished by abolished NaVO_3_-induced VSMC IL-6 and VSMC migration (Figure 4D,E). These results indicate that ROS derived by NADPH oxidases contributes to NaVO_3_-induced cytosolic ROS production and VSMC pathological responses.

Given that ROS and IL-6 are involved in NaVO_3_-induced VSMC proliferation and migration, we thus determined a pathological connection between ROS and IL-6. NAC significantly dose-dependently reduced NaVO_3_-induced IL-6 production (Figure 4F). Conversely, anti-IL-6 neutralizing antibodies did not affect NaVO_3_-induced intracellular ROS production (Figure 4G). These results indicate that NaVO_3_-induced IL-6 production is mediated through intracellular ROS and the ROS-mediated IL-6 is required for NaVO_3_-induced VSMC proliferation and migration. 

### 2.5. Involvement of Signaling Kinases in NaVO_3_-Mediated VSMC Migration

**Figure 5:**
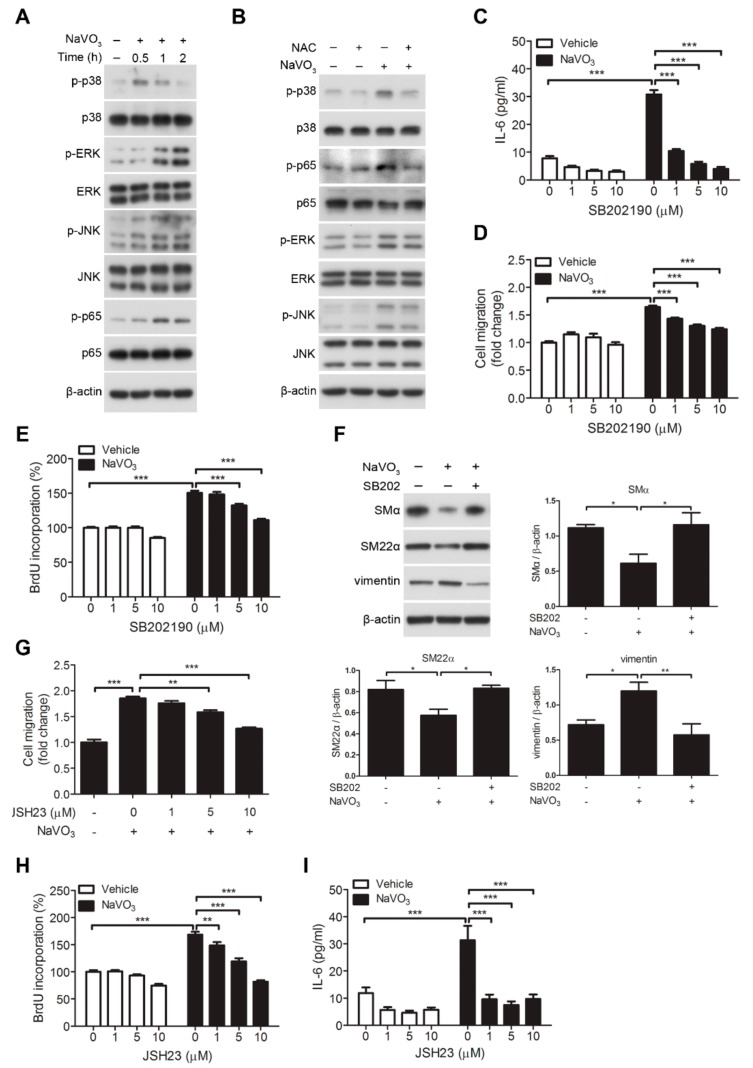
p38 MAPK-mediated NF-κB signaling is essential for NaVO_3_-mediated responses in VSMCs. (**A**) VSMCs were treated with NaVO_3_ (1 µg/mL) for the indicated times. (**B**) After pretreating VSMCs with NAC (1 mM) for 30 min, cells were stimulated with or without NaVO_3_ (1 µg/mL) for 1 h. Cell lysates were immunoblotted with antibodies for p38 MAPK, phosphor-p38 MAPK, ERK1/2, phosphor-ERK1/2, JNK1/2, phosphor-JNK1/2, NF-κB p65, phosphor- NF-κB p65, or β-actin. The experiments were repeated three times with similar results. (**C**–**E**) VSMCs were treated with vehicle (DMSO) or NaVO_3_ with different concentrations of SB202190 (1–10 µM) for 24 h. (**C**) IL-6 levels in culture supernatants were measured by ELISA. (**D**) VSMC migration was measured by the transwell assays. (**E**) VSMC proliferation was measured by BrdU incorporation assay. (**F**) VSMCs were treated with DMSO or NaVO_3_ with SB202190 (10 µM, SB202) for 48 h. Cell lysates were immunoblotted with antibodies for SMα, SM22α, vimentin or β-actin. Densitometry analysis of SMα, SM22α, and vimentin protein expression relative to β-actin. (**G**–**I**) VSMCs were pretreated with different concentrations of JSH23 (1–10 µM) for 30 min, then stimulated with NaVO_3_ for 24 h. (**G**) VSMC migration was then measured by the transwell assays. (**H**) VSMC proliferation was measured by BrdU incorporation assay. (**I**) IL-6 levels in culture supernatants were measured by ELISA. Data represent mean ± SEM of three experiments. * *p* < 0.05; ** *p* < 0.01; *** *p* < 0.001.

Our results revealed that NaVO_3_, but not the vehicle control, strongly induced the phosphorylation of p38 MAPK (mitogen-activated protein kinase), ERK1/2 (extracellular signal–regulated kinases 1/2), JNK1/2 (c-Jun NH_2_-terminal kinase 1/2), and NF-κB p65 in VSMCs (Figure 5A). Inhibition of ROS signaling by NAC significantly abolished NaVO_3_-induced phosphor-p38 and phosphor- NF-κB p65 but not phosphor-ERK1/2 and phosphor-JNK1/2 (Figure 5B). Furthermore, we used pharmacological inhibitors to reveal that p38 MAPK inhibitor (SB202190), ERK1/2 inhibitor (U0126), and JNK inhibitor (SP600125) had no significant effect on cell viability (Figure S3C–E) and NaVO_3_-induced ROS production (Figure S4A–C). These results suggest that ROS is upstream of p38 in NaVO_3_-driven signaling pathways. We next wondered if the p38 participated in NaVO3-mediated functional responses in VSMCs. Our results showed that NaVO_3_-induced VSMC IL-6 secretion and VSMC migration and proliferation were suppressed by SB202190 (Figure 5C–E). Furthermore, this p38 inhibitor abrogated NaVO_3_-mediated reduction of SMα and SM22α and enhancement of vimentin in VSMCs (Figure 5F). Activation of NF-κB mediated by p38 MAPK is known to play an important role in the regulation of VSMC IL-6 secretion and control of VSMC function [32]. We thus evaluated the effect of NF-κB inhibitor JSH23 on NaVO_3_-mediated VSMC function such as migration and proliferation and IL-6 production. JSH23 significantly blocked NaVO_3_-induced VSMC migration and proliferation (Figure 5G,H) and IL-6 production but not ROS generation in the VSMCs (Figure 5I and Figure S4D) without affecting VSMC viability (Figure S3F). Collectively, these results imply that NaVO_3_ induces ROS to activate p38 MAPK signaling, thereby triggering NF-κB-mediated IL-6 production, which subsequently promotes VSMC migration and proliferation.

### 2.6. Anti-Oxidant N-Acetylcysteine Prevents NaVO_3_-Induced Atherosclerosis in ApoE^−/−^ Mice

**Figure 6:**
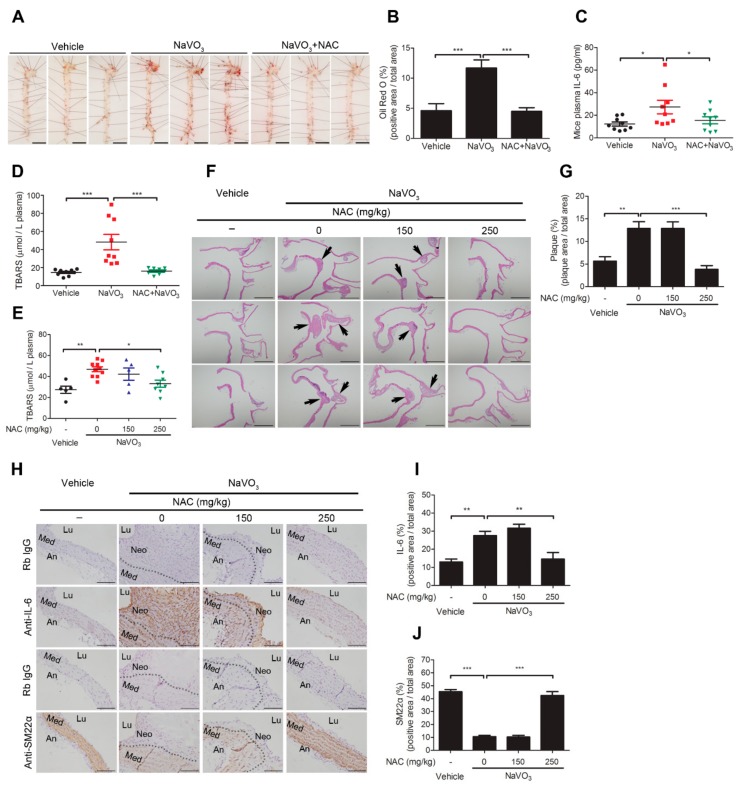
Anti-oxidant *N*-acetylcysteine prevents NaVO_3_-induced atherosclerosis in *ApoE^−/−^* mice. *ApoE^−/−^* mice administered NaVO_3_ (4 mg/kg) once a week were injected intraperitoneally with saline (vehicle) or NAC (250 mg/kg) three times weekly for 12 weeks (*n* = 9 vehicle; *n* = 9 NaVO_3_; *n* = 8 NaVO_3_ + NAC). (**A**) Lipid contents in aorta were analyzed by Oil Red O staining. Scale bars represent 5 mm. (**B**) The area of positive staining for Oil Red O was quantified using ImageJ software as a percentage of total aortic area. (**C**) Plasma IL-6 levels were measured by ELISA. (**D**) Plasma ROS levels were measured by TBARS assay. (**E**–**J**) *ApoE^−/−^* mice administered NaVO_3_ (4 mg/kg) once a week were injected intraperitoneally with different amounts of NAC (150 or 250 mg/kg) three times weekly for 12 weeks (*n* = 5 vehicle; *n* = 10 NaVO_3_; *n* = 5 NaVO_3_ + 150 mg/kg NAC; *n* = 8 NaVO_3_ + 250 mg/kg NAC). (**E**) Plasma ROS levels were measured by TBARS assay. (**F**) Paraffin-embedded tissues with atherosclerotic plaque (black arrow) were observed by hematoxylin and eosin stain at 40× magnification. Scale bars represent 1 mm. (**G**) Atherosclerotic plaque in paraffin-embedded aorta tissues were quantified using ImageJ software as a percentage of total aortic area in each section. (**H**) IL-6 and SM22α contents in aorta tissues were analyzed by immunohistochemistry. Immunopositive areas are shown at 400× magnification. An: adventitia; Lu: lumen; Neo: neointima; Med: media. Negative control represents staining with an isotype control antibody. Scale bars represent 100 µm. (**I**) IL-6 and (**J**) SM22α immunopositive areas in paraffin-embedded aorta tissues were quantified using ImageJ software as a percentage of total aortic area in each section. Data represent mean ± SEM. The solid black line denotes the mean value. * *p* < 0.05; ** *p* < 0.01; *** *p* < 0.001.

Induction of VSMC ROS generation by NaVO_3_ could contribute to excessive plasma ROS and IL-6 and consequent atherosclerotic lesions. We thus determined whether exogenous anti-oxidant *N*-acetylcysteine administration reduces plasmas ROS and IL-6 levels induced by NaVO_3_ and rescues mice from NaVO_3_-induced atherosclerosis. *ApoE^−/−^* mice exposed to NaVO_3_ were injected with NAC (250 mg/kg) or vehicle three times a week for 12 weeks and atherosclerotic lipid accumulation and plasma ROS and IL-6 were measured. Mice treated with NAC suppressed NaVO_3_-induced vascular lipid accumulation and plasma IL-6 production (Figure 6A–C), correlating with a significant decrease in plasma ROS (Figure 6D). To further confirm the protective effect of NAC on atherosclerosis, the suppression of atherosclerotic pathological alternations was evaluated in the NaVO_3_-exposed mice administered with a different dose of NAC (150 and 250 mg/kg). The results suggest that although NAC had no effect on vanadium level of urine (Figure S5A), it not only dose-dependently reduced NaVO_3_-induced plasma ROS but also inhibited NaVO_3_-induced atherosclerotic plaque and aortic lipid accumulation when compared with vehicle treatments (Figure 6E–G and Figure S5B), which concurrently attenuated lung injury (Figure S5C). In addition, NaVO_3_-mediated IL-6 enhancement and SM22α reduction in the atherosclerotic lesions were suppressed by NAC in a dose-dependent manner (Figure 6H–J), indicating that NaVO_3_ triggers ROS-meditated IL-6 production to induce atherosclerosis.

## 3. Discussion

Although the causes of vascular diseases are complex, it is being recognized that environmental factors such as PM can play a significant role [6,33]. Metals in PM are considered to contribute to the particle’s toxic effects. Numerous epidemiological studies link PM metal exposure to an increased risk of vascular diseases and cardiovascular mortality [5,6]. Vanadium is classified as a group 5 transition metal and is a redox-sensitive element. It exists in a large number of oxidation states, though the V(IV) and V(V) oxidation states are most common under physiological conditions. Vanadium pentoxide is considered an air toxicant with mutagenic effects, respiratory tract toxicity, and possible carcinogenic activity [11,13,34,35]. Despite much evidence indicating the pathological toxic effects of vanadium oxide on health, its pathological influence and mechanism on atherosclerosis remain unclear. In this study, we used *ApoE^−/−^* mice and found that exposure to NaVO_3_ not only causes lung injury, consistent with previous reports, but also induces atherosclerotic plaque. The NaVO_3_-exposed mice developed downregulation of VSMC marker SM22α and upregulation of atherosclerotic-promoting cytokine IL-6 within atherosclerotic lesions. In addition, these atherosclerotic pathologies were accompanied by increasing plasma ROS and IL-6. Notably, these NaVO_3_-induced atherosclerotic pathologies in *ApoE^−/−^* mice were prevented by NAC treatment, indicating that ROS is required for NaVO_3_-driven atherosclerosis. Our results further show that VOSO_4_ and NaVO_3_ promote phenotypic transitions of VSMCs from the quiescent contractile state to the active synthetic state and VSMC proliferation and migration, which has been implicated in deleterious consequences of atherosclerosis [3,25,26,29]. The present study provides experimental evidence that vanadium salts, VOSO_4_ and NaVO_3_, drive VSMC pathological responses by inducing ROS production and thereby activating p38/NF-κB signaling. Our results provide new insights into the pathological mechanism underlying VOSO_4_- and NaVO_3_-induced atherosclerosis and VSMC dysfunction.

Under physiological conditions, ROS function as signaling molecules, playing a vital role in maintaining vascular homeostasis and prevent cardiovascular inflammation and injury [31]. However, during pathological stress, aberrant concentrations of ROS in the vessel wall are produced and disturb the redox homeostasis and subsequently influence the function of vascular cells such as VSMCs. Given that transition metals with biological toxicity are due to the formation of oxidizing compounds that exert their toxic effects by the production of ROS [5,36], we propose that oxidizing vanadium compound induces ROS overproduction and activates ROS-mediated atherosclerotic signaling. This hypothesis is supported by anti-oxidant *N*-acetylcysteine (NAC) rescue of *ApoE^−/−^* mice from NaVO_3_-induced excessive plasma ROS and IL-6 and consequent atherosclerosis. In addition, NaVO_3_-driven VSMC pathological effects including ROS and IL-6 production, phenotypic alternation, migration, and proliferation are suppressed by NAC. These suggest that NaVO_3_ increases vascular cell ROS production thereby increasing the plasma ROS level and mediating VSMC dysfunction and atherosclerosis.

Although ROS are generated from a number of sources including NADPH oxidases, xanthine oxidase, the mitochondrial respiratory chain, lipoxygenases, and nitric oxide synthases, the mitochondria and NADPH oxidases have been considered to be the major sources of ROS in VSMCs [21,37]. Here, we have provided experimental evidence that ROS derived from NADPH oxidases, but not mitochondria, are critical mediators of NaVO_3_-triggered VSMC pathological effects. Our results show that NaVO_3_ significantly increases intracellular ROS but negligibly increases mitochondrial ROS production. However, NADPH oxidase inhibitor significantly reduces NaVO_3_-induced ROS generation and IL-6 production. These findings indicate that NADPH oxidases are a major source of ROS that contribute to NaVO_3_-mediated pathological signaling in VSMCs.

The pleiotropic cytokine IL-6 is a proatherogenic factor that is increased in atherosclerotic mouse plasma and in VSMCs in response to atherosclerotic stimulators. Accumulating evidence suggests IL-6 is implicated in the progression of atherosclerosis and plays a key role in inducing VSMC migration and proliferation [25,26,38,39]. Our findings indicate that NaVO_3_ triggers VSMC migration and proliferation through ROS-mediated IL-6 production. This notion is supported by abrogation of NaVO_3_-induced VSMC IL-6 production, VSMC migration, and VSMC proliferation by NAC and NADPH oxidase inhibitor without affecting VSMC viability. We further show that anti-IL-6 antibodies suppressed NaVO_3_-induced VSMC migration and proliferation (Figure 2G–H) but IL-6 antibodies did not affect VSMC ROS production (Figure 4G). These results suggest that ROS-mediated IL-6 secretion induced by NaVO_3_ acts in an autocrine or paracrine manner to regulate VSMC pathological function and thereby modulate atherosclerosis development. Although ROS-driven cellular signaling are reported to play an important role in controlling cell function and disease progression [40], little is known about the regulation of oxidizing vanadium derivative-modulated ROS-mediated IL-6 expression in VSMCs. In the present study, we found that NaVO_3_ increased the phosphorylation level of p38, ERK, JNK, and NF-κB p65, but inhibiting ROS signaling by NAC only suppressed the levels of phosphor-p38 and phosphor-NF-κB p65. Furthermore, inhibition of p38 and NF-κB signaling by their pharmacological inhibitors significantly abolished NaVO_3_-induced VSMC migration and proliferation and IL-6 production without affecting ROS production in VSMCs. These results suggest that NaVO_3_ promotes VSMC phenotypic switch, migration, and proliferation by increasing IL-6 production, which is mediated via ROS-activated p38/NF-κB-dependent signaling. 

Vanadium is widely distributed throughout the Earth’s crust at concentrations of 100–150 ppm (approximately 100–150 mg/kg) [41,42]; greater levels in urban locations have been reported and may reach higher values (up to 400 ppm) in areas polluted by fly ash due to a larger density of combustion sources capable of emitting PM-containing vanadium to the environment. Much evidence from epidemiological and toxicological studies suggests that vanadium exposure causes serious health problems such as lung disease, eye irritation, and hypertension in humans [43,44], but its toxic effects on vascular diseases remains unclear. In this study, we used an animal model of *ApoE^−/−^* mice to demonstrate that vanadium exposure has detrimental effects on increasing plasma ROS and atherosclerotic cytokine IL-6 and consequently promotes the synthetic phenotype in VSMCs, which leads to atherosclerosis. We provide evidence that the oxidizing vanadium salts, VOSO_4_ and NaVO_3_, promote VSMC synthetic differentiation, migration, and proliferation and consequent atherosclerosis via ROS-mediated IL-6 induction. These findings provide mechanistic insights into the key vascular pathological role of VOSO_4_- and NaVO_3_-mediated ROS signaling in VSMC dysfunction and atherosclerosis as demonstrated by the anti-oxidant NAC, which not only suppresses VSMC pathological responses including migration and proliferation but also prevents atherosclerosis in *ApoE^−/−^* mice. Our results suggest that ROS-mediated IL-6 signaling will be a valuable therapeutic target to prevent vanadium air pollution-mediated inappropriate smooth muscle cell function and atherosclerosis.

Our findings indicate that the oxidizing vanadium salts, VOSO_4_ and NaVO_3,_ are capable of mediating VSMC migration and proliferation which lead to the development of atherosclerosis. We have provided evidence that these two vanadium salts promote VSMC migration and proliferation by activating NF-κB-mediated IL-6 production, which is signaled via ROS-dependent activation of the p38 MAPK. These findings provide mechanistic insights into the pathological effect of VOSO_4_ and NaVO_3_ in atherosclerosis and will be valuable for developing new therapeutic strategies against vascular diseases.

## 4. Materials and Methods

### 4.1. Materials 

Sodium trioxovanadate (NaVO_3_), oxovanadium sulfate (VOSO_4_), SB202190, SP600125, and 6-carboxy-2,7-dichlorodihydrofluorescein diacetate (DCFDA) were purchased from Sigma-Aldrich (St. Louis, MO, USA). U0126 and MitoSOX Red fluorescence were from InvivoGen (San Diego, CA, USA) and Molecular Probes (Invitrogen, Carlsbad, CA, USA), respectively. Antibodies for E-cadherin (610182, BD) SMα (A5228, Sigma-Aldrich), SM22α (ab14106, Abcam, Cambridge, MA, USA), vimentin (550513, BD Biosciences, San Jose, CA, USA), β-actin (MAB1501, Millipore, MA, USA)*,* IL-6 (ab6672, Abcam), p-p38 (#4511, CST), p38 (sc-7972, Santa Cruz, CA, USA), p-ERK (sc-7383, Santa Cruz), ERK (sc-94, Santa Cruz), p-JNK (#9251, Cell Signaling Danvers, MA, USA), JNK (#9258, Cell Signaling), p-p65 (Cell Signaling #3033), and p65 (#4764, Cell Signaling) were used in western blot analysis and immunohistochemistry.

### 4.2. Preparation of NaVO3 and VOSO4 Solutions

NaVO3 (4 mg/mL) solution was freshly prepared by dissolving the required amounts of NaVO_3_ in endotoxin-free water. Briefly, NaVO_3_ was suspended in endotoxin-free water, heated at 55 °C for 20 min and vortex every 10 min until dissolved in endotoxin-free water. Before the experiment, the solution was allowed to stand at room temperature with a pH of about 6.8.

To prepare the VOSO_4_ solution, the VOSO_4_ was dissolved in the DMEM at a working concentration of 1 µg/mL (pH about 8.2~8.6).

### 4.3. Animals

Wild-type C57BL/6J mice (National Laboratory Animal Center, Taiwan) were used for primary vascular smooth muscle cell (VSMC) isolation, and *ApoE^−/−^* mice (C57BL/6 background, Jackson Laboratory, Bar Harbor, ME, USA) (8–12 weeks old) were randomly grouped to experiments and fed normal chow. Mice were housed in a specific pathogen-free animal facility at National Health Research Institutes, Taiwan. All experimental procedures were followed by the NIH guidelines for the laboratory animal’s care and use and approved by the Institutional Animal Care and Use Committee of National Health Research Institutes, Taiwan (#NHRI-IACUC-106081-A, 02/01/2017).

### 4.4. Induction of Atherosclerosis in a Mouse Model

Eight-to-twelve-week-old *ApoE^−/−^* mice (C57BL/6 background, Jackson Laboratory) were randomly assigned and intranasally administered vehicle (endotoxin-free water) or NaVO_3_ (4 mg/kg) once weekly with or without intraperitoneal injection of NAC (150 mg/kg or 250 mg/kg) three times a week for 12 weeks. This method followed the guidelines of experimental atherosclerosis studies described in the AHA (American Heart Association) Statement [45]. Following this, the mice were killed, and blood samples were collected for vanadium, ROS, and cytokine analysis. Mouse urine was collected on week 4 and 6 for urine vanadium level measurements. The aortas were harvested for analysis of atherosclerotic lesion. In addition, the lung, kidney, liver, and heart were collected for histological determination. To be the more specific in terms of the measurement of the atherosclerotic area in mouse lesion sites from the ascending aorta to the end of the arch, we made three 4-µm sections on each slide, with a total of 100 slides in a sequential manner. We picked one slide from every 10 slides and 10 slides for each group to perform H&E (hematoxylin and eosin) or immunohistochemistry (IHC) staining. The mouse experiments were approved by the Institutional Animal Care and Use Committee, National Health Research Institutes.

### 4.5. Cell Culture and Treatment

Primary VSMCs were isolated from 18.5-day-postconception embryonic mouse aortas of C57BL/6J mice (The National Laboratory Animal Center, Taipei, Taiwan) by enzymatic digestion of the aorta using collagenase and elastase as previously described [25]. In briefly, VSMCs were cultured in a growth medium containing Dulbecco’s Modified Eagle medium (DMEM) supplemented with 10% FBS, penicillin (100 U/mL), and streptomycin (100 μg/mL) as previously described. Cells were passaged every 3 days, and experiments were performed on cells 5–10 passages after the primary culture. VSMCs were quiescent by starvation in 0.5% FBS in DMEM for 24 h before experiments.

VSMCs were pre-incubated with or without inhibitors for 30 min before treatment with endotoxin-free water, NaVO_3_, or VOSO_4_ (1–5 μg/mL) in DMEM supplemented with 10% FBS, penicillin (100 U/mL), streptomycin (100 μg/mL), 50 mg/L l-ascorbic acid, and 2.16 g/L β-glycerophosphate (Sigma-Aldrich) for the indicated time, unless specified otherwise.

To investigate the effect of IL-6 on NaVO_3_-induced VSMC proliferation and migration, VSMCs were treated with NaVO_3_ (1μg/mL) with or without rat-IgG (2.5 μg/mL), anti-IL-6 (2.5 μg/mL) antibodies (eBioscience, San Diego, CA, USA) for the indicated time.

### 4.6. Measurement of Vanadium Levels in Urine and Plasma

The vanadium concentrations in the urine and plasma were measured by inductively coupled plasma–mass spectrometry (ICP-MS, NexION 2000, PerkinElmer, Waltham, MA, USA). Before measurement, all the samples were diluted 1:10 with 70% nitric acid and microwave digestion to provide clean samples. All samples were quantified using linear calibration curve established from the analysis of a series of five standard solutions (0.1, 0.5, 1, 5, and 10 parts per billion (ppb)) with typical correlation coefficients (R^2^) greater than 0.9999 for each analysis to ensure that the measurement process was reliable. The vanadium concentrations in the plasma samples were expressed as nanograms per milliliter (ng/mL); whereas the levels of vanadium in urine were normalized by creatinine (CRE) in the urine sample and expressed as micrograms per gram creatinine (μg/g CRE).

### 4.7. TBARS Assay

The plasma ROS was determined by measurement of lipid peroxidation via thiobarbituric acid reactive substances (TBARS) assay (Cayman Chemical, Ann Arbor, MI, USA) according to manufacturer’s protocol. The concentration of TBARS in plasma were calculated from TBARS standard curve (0 to 400 μmol/L 1,1,3,3-tetramethoxypropane) and was expressed as µmol/L.

### 4.8. Oil Red O Staining

The aorta was dissected of connective tissue under a dissecting microscope and stained with 0.9% Oil Red O (in 66.6% isopropanol) solution for 15 min at room temperature. After destaining with 66.6% isopropanol for 10 min (three times), the aorta was fixed on dissection disk with an insect needle and inspected under a microscope.

### 4.9. Histology and Immunohistochemistry 

For histological studies, mice were perfused with saline and subsequently with 10% formaldehyde. The tissues, including lung, liver, kidney, heart, and aorta, were immersed in formaldehyde for 24 h and then dehydrated with a graded series of ethanol and embedded in paraffin. Tissue blocks were cut into 4-µm-thick serial sections and stained with hematoxylin and eosin for examining the pathology, such as atherosclerotic plaque and lung inflammation and injury.

Prior to detection of tissue protein expression by immunohistochemistry, the aortic sections were deparaffinized with xylene and progressively rehydrated through graded alcohols. Antigen retrieval were performed by heating the sections on slides in an EDTA antigen retrieval buffer of pH 8 (Trilogy; Cell Marque Corporation) in an electric pressure cooker for 10 min. Sections were sequentially blocked by 3% H_2_O_2_ for 20 min and blocking buffer (5% BSA in phosphate-buffered saline with 0.1% Tween 20 (PBST)) for an additional 30 min. Antibodies including SM22α (ab14106, Abcam) and IL-6 (ab6672, Abcam) antibodies were diluted in PBS. Sections were incubated at room temperature for 2 h or 4 °C for overnight with primary antibody and then washed in PBST. The sections were then incubated with HRP Labelled Polymer (Dako, Carpinteria, CA, USA) for 60 min and washed three times with PBST. The protein expression was visualized using the DAB Chromogen system (Dako). To validate the specificity of primary antibody, there was a negative control representing staining with an isotype control antibody. Slides were counterstained with hematoxylin. The immunopositive areas in the aorta tissues were quantified using ImageJ software. The signal values were expressed as the percent positive area out of the total tissue area.

### 4.10. Western Blot Analysis

Cellular proteins extracted from treated VSMCs were resolved with 4–12% SDS-PAGE and transferred to the polyvinylidene difluoride (PVDF) membranes [46]. 

### 4.11. Cell Viability and Cell Proliferation Assays

MTT assay was used to measure cell viability. Cell proliferation was determined by bromodeoxyuridine (BrdU) incorporation assays. Briefly, VSMCs were plated on microtiter plates in starvation medium for 24 h and treated with different amounts of VOSO_4_ or NaVO_3_ for the indicated times. For MTT assay, the treated VSMCs were incubated DMEM containing 0.5 mg/mL thiazolyl blue tetrazolium bromide (MTT) for 1 h, then lysed by DMSO. The formazan reduced by mitochondrial dehydrogenases of living cells were read by microplate reader at absorbance 540 nm. BrdU was added directly to cell cultures for 24 h starting at time of stimulation. The BrdU incorporation assays was performed according to the manufacturer’s protocol (Millipore).

### 4.12. Migration Assays

To assess migration, quiesced VSMCs were treated with or without VOSO_4_ or NaVO_3_ (1 µg/mL) with or without inhibitor for 24 h. VSMCs were trypsinized, washed with PBS, re-suspended in medium with 0.5 % FBS, and then placed in the upper chamber of 24-well transwell plates (Millipore, 8-μm pore size) in triplicate (20,000 cells/well). The bottom chambers were filled with starvation medium containing PDGF-BB (Peprotech, 10 ng/mL) as a chemoattractant. After 4 h, the upper layer was scraped by cotton swab to remove non migratory cells, membrane fixed, and stained with crystal violet solution (0.1% crystal violet, 20% ethanol, and 1% formaldehyde in ddH_2_O). Cells that had migrated to the underside of the membrane were visualized by microscope. The crystal violet in the cells was extracted by crystal violet extraction buffer (50% ethanol and 0.1% acetic acid in ddH_2_O) and read by a microplate reader at absorbance 595 nm. The data were plotted as the fold change versus vehicle, arbitrarily set to 1.

### 4.13. IL-6 ELISA

IL-6 levels in the plasma and culture supernatants were determined in microtiter plates (96-well) by a mouse IL-6 ELISA (eBioscience; 14-7061-85 and 13-7062-85) as previously described [47].

### 4.14. Cellular ROS and Mitochondria ROS Assay

Intracellular ROS or mitochondria ROS (superoxide) were measured using cell-permeable dyes 2,7-dichlorofluorescein derivative 6-carboxy-2,7-dichlorodihydrofluorescein diacetate (DCFDA) or MitoSOX Red fluorescence, respectively. After pretreating VSMCs with different concentrations of inhibitor for 30 min, cells were stimulated with or without NaVO_3_ (1 µg/mL) for 24 h. VSMCs were then stained with 10 µM DCFDA for 15 min or 5 µM MitoSOX for 10 min in DMEM. After washing with PBS three times, the cells were suspended in PBS and analyzed by flow cytometry.

### 4.15. Statistical Analysis

Statistical analyses were performed using Graphpad Prism version 5 Software (GraphPad Software Inc., San Diego, CA, USA). All values were given as means ± SEM. The *t*-test (two-tailed) was used to determine the statistical significance of the difference between the vehicle and treatment groups. While analyzing multiple groups, one-way ANOVA with multiple comparisons test (Tukey’s, Bonferroni’s, Newman–Keuls’s) or two-way ANOVA with multiple comparisons test (Bonferroni’s) were used and *p* values < 0.05 were considered statistically significant.

## Supplementary Materials

Supplementary materials can be found at https://www.mdpi.com/1422-0067/20/24/6115/s1.

## Abbreviations

VSMC	Vascular smooth muscle cell
NAC	*N*-acetylcysteine
ROS	Reactive oxygen species
FBS	Fetal bovine serum (FBS)
