# Supplementary material for: Vanadium Derivative Exposure Promotes Functional Alterations of VSMCs and Consequent Atherosclerosis via ROS/p38/NF-κB-Mediated IL-6 Production
Source: Int J Mol Sci. 2019 Dec 4;20(24):6115. doi: 10.3390/ijms20246115 (PMC6940940; doi:10.3390/ijms20246115)
Supplement: Supplementary file 1 [file ijms-20-06115-s001.pdf]

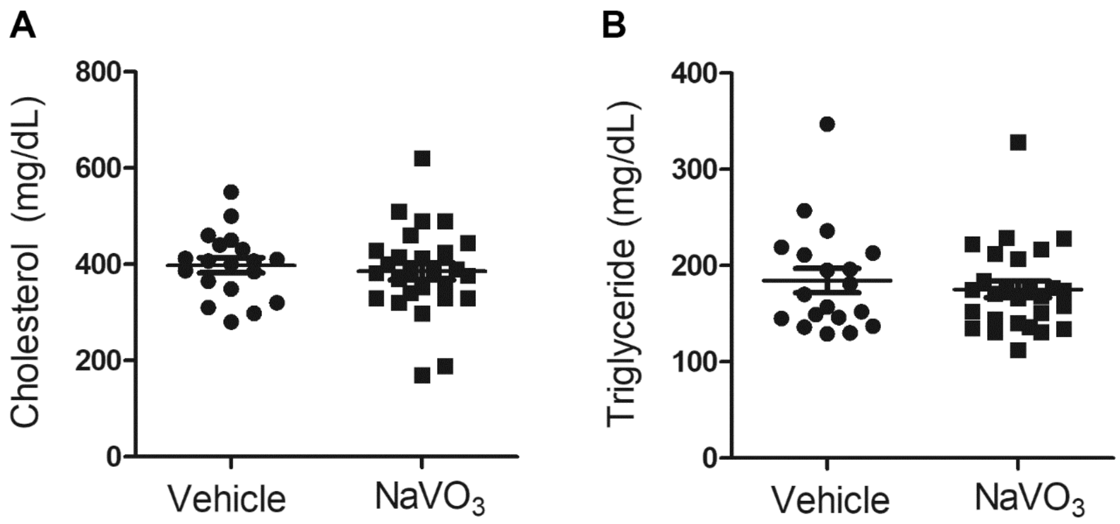

Figure S1. Effect of vanadium oxide on plasma lipid. ApoE$^{-/-}$ mice were intranasal administrated with vehicle (endotoxin free water) or $NaVO_3$ (4 mg/kg) once a week for 12 weeks. Plasma from ApoE$^{-/-}$ mice were treated with vehicle (n = 19) and $NaVO_3$ (n = 27). (A) The cholesterol and (B) triglyceride were measured by DRI-CHEM 3500s biochemistry analyzer.

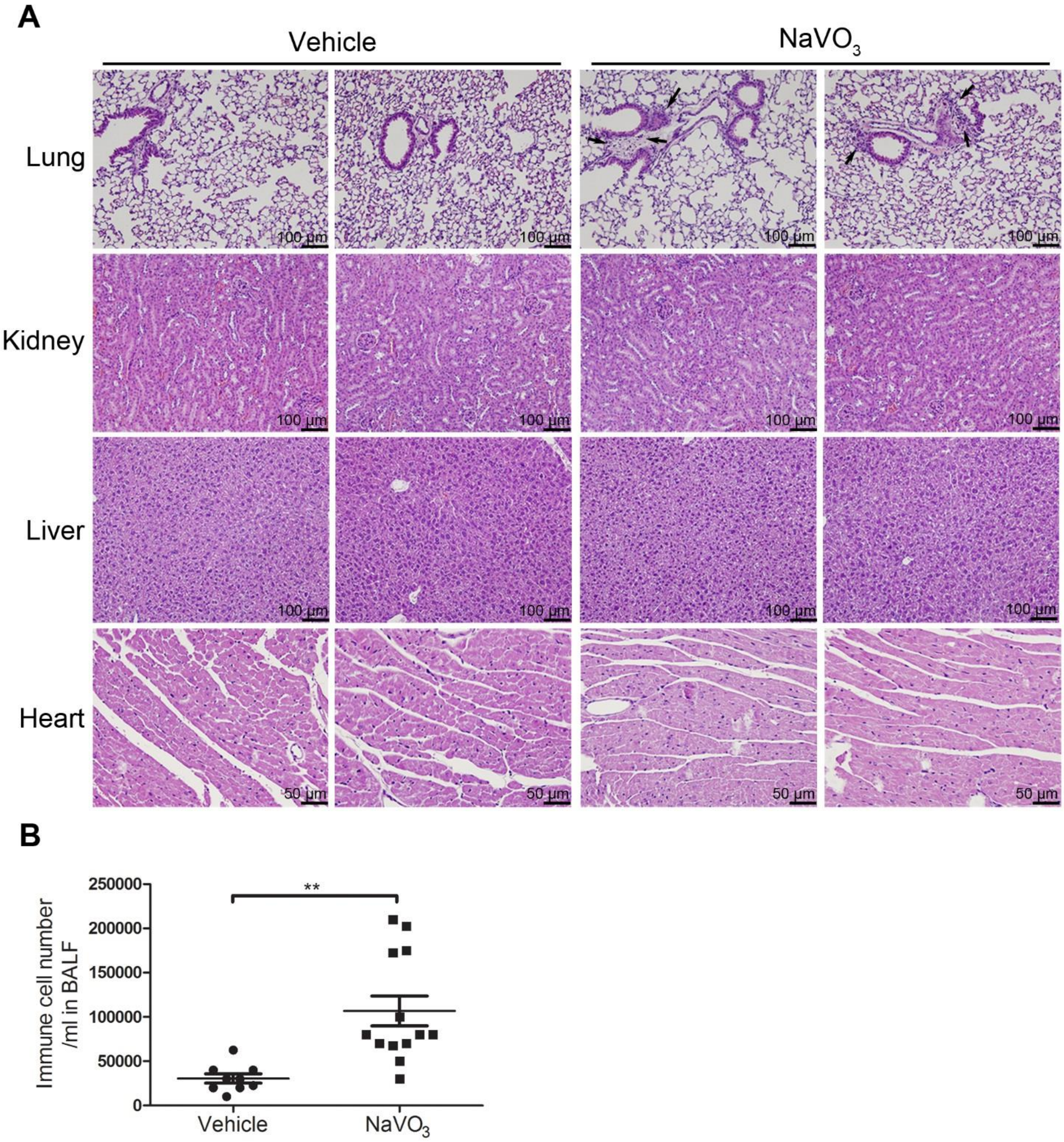

Figure S2. Intranasal administration of $NaVO_3$ induced immune cells infiltration into lung. (A) Representatively hematoxylin and eosin (H&E) staining of lung, liver, kidney, and heart tissue from ApoE$^{-/-}$ mice intranasal administrated with vehicle (endotoxin free water, n = 5) or $NaVO_3$ (n = 5) once a week for 12 weeks. Sections were examined and photographed under a microscope. Black arrows indicate infiltrated immune cells. (B) Total cell number in bronchoalveolar lavage fluid (BALF) of vehicle (endotoxin free water, n = 9) or $NaVO_3$ (n = 13) treated ApoE$^{-/-}$ mice was determined by trypan blue exclusion assay. **$P < 0.01$.

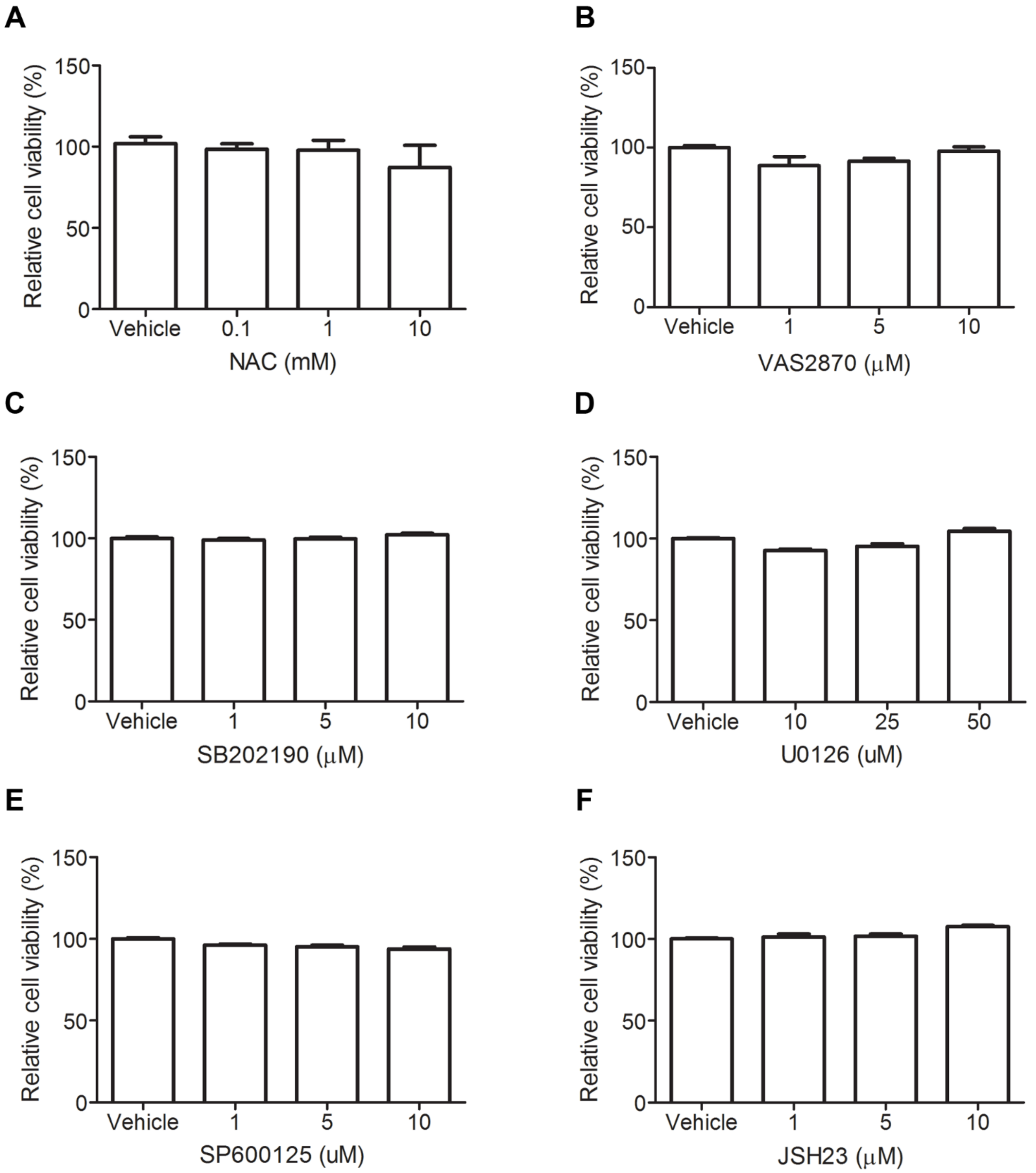

Figure S3. Effect of anti-oxidant N-acetylcysteine, pan-NADPH oxidase inhibitor (VAS2870), p38 MAPK inhibitor (SB202190), ERK1/2 inhibitor (U0126), JNK inhibitor (SP600125), and NF-κB p65 inhibitor (JSH23) on VSMC viability. Quiescent VSMCs were treated with the indicated concentration of (A) N-acetylcysteine (NAC), (B) VAS2870, (C) SB202190, (D) U0126, (E) SP600125, or (F) JSH23 for 24 h, then MTT assay was performed to determine cell viability.

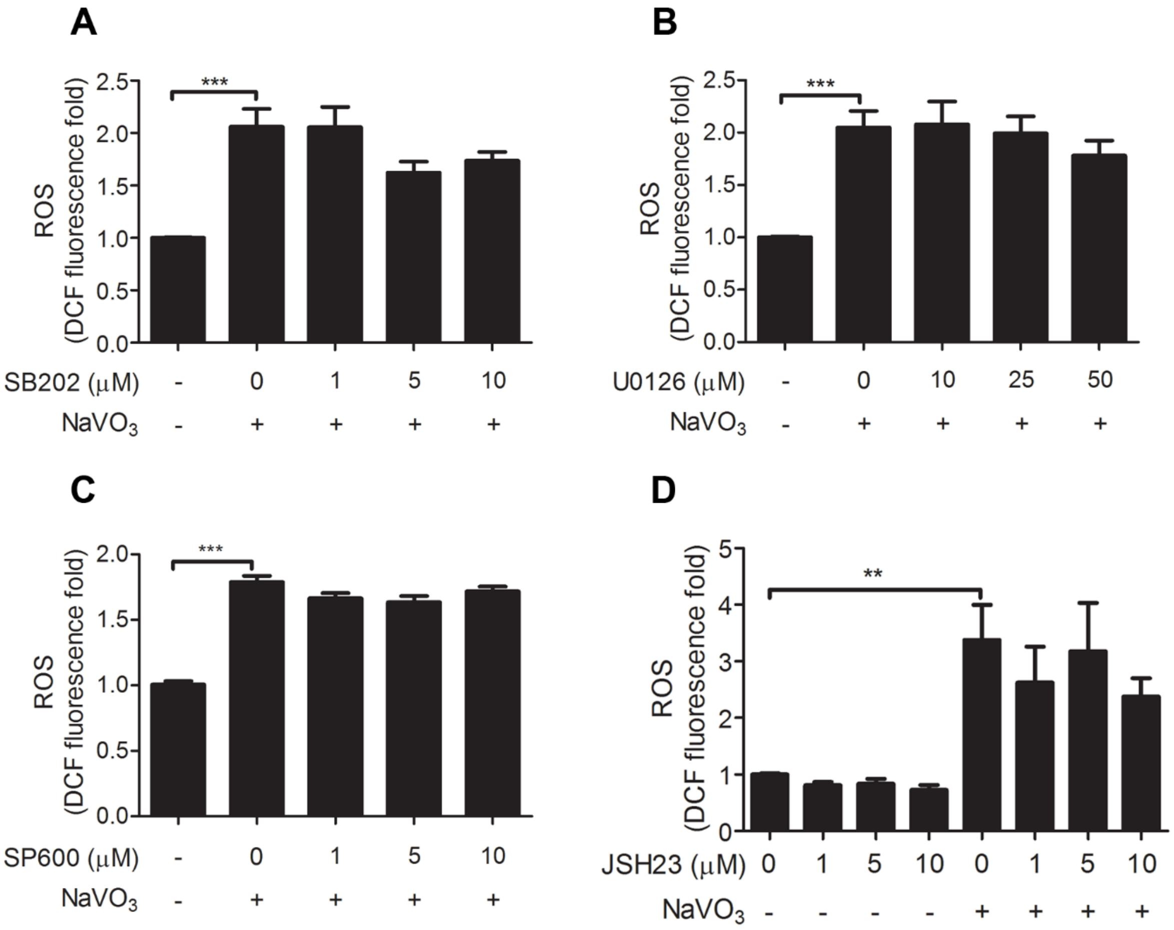

Figure S4. Effect of MAPK kinase inhibitors and NF-κB p65 inhibitor on $NaVO_3$- induced ROS in VSMCs. VSMCs were pretreated with different concentrations of (A) SB202190 (1–10 μM, SB202), (B) U0126 (10–50 μM), (C) SP600125 (1–10 μM, SP600) or (D) JSH23 (1–10 μM) for 30 min, then stimulated with or without $NaVO_3$ (1 μg/ml) for 24 h. Cellular ROS were measured using DCFDA staining and quantified by flow cytometry. $^{**}P < 0.01$; $^{***}P < 0.001$.

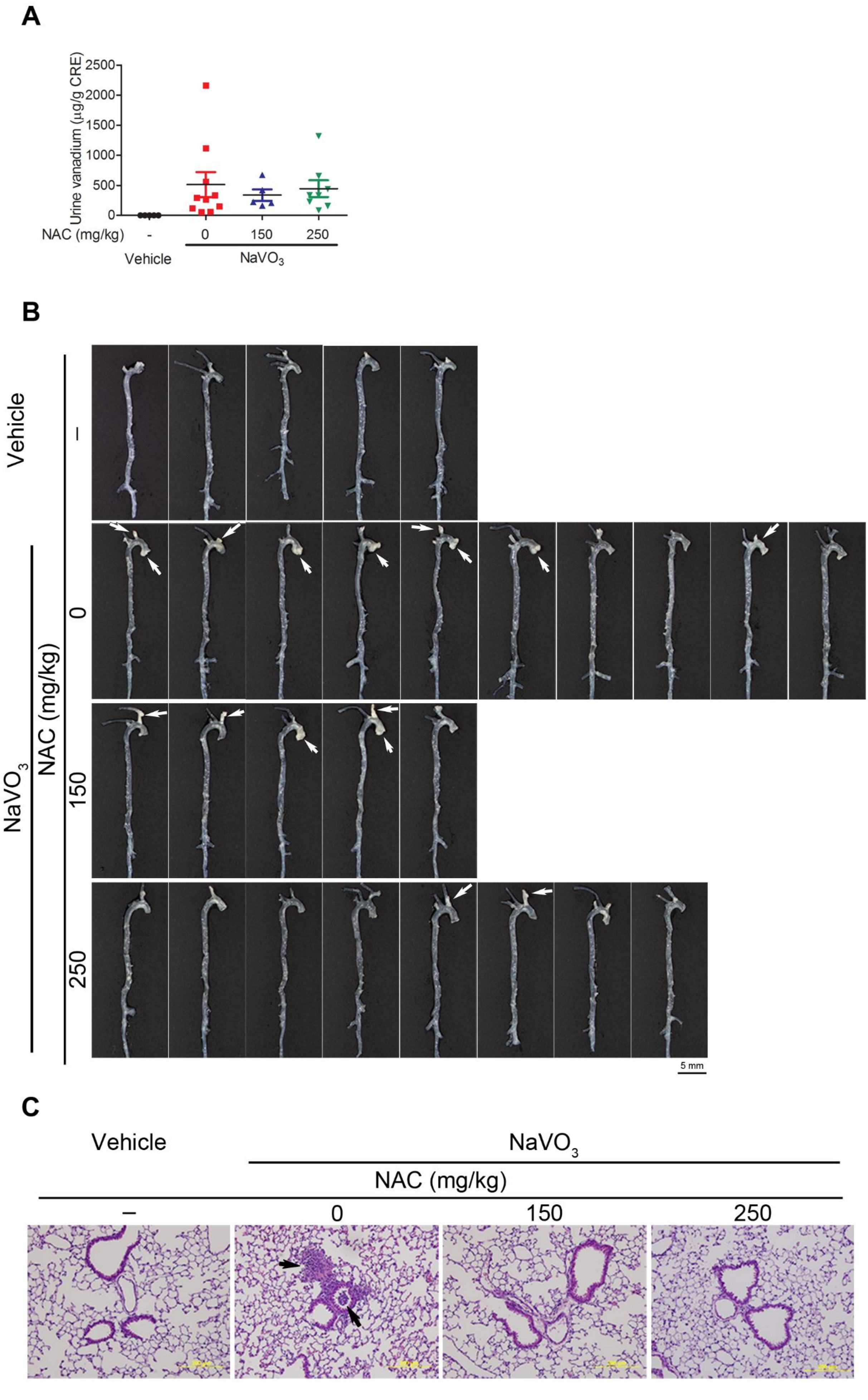
A
Urine vanadium (μg/g CRE)
2500
2000
1500
1000
500
0
NAC (mg/kg)
-
0
150
250
Vehicle
NaVO3
B
Vehicle
NaVO3
NAC (mg/kg)
–
0
150
250
5 mm
C
Vehicle
NaVO3
NAC (mg/kg)
–
0
150
250

Figure S5. Anti-oxidant N-acetylcysteine prevents $NaVO_3$-induced lung injures and atherosclerosis in ApoE$^{-/-}$ mice, but no effect on vanadium level in urine. ApoE$^{-/-}$ mice were intranasal administrated with vehicle (endotoxin free water) or $NaVO_3$ once a week were followed by intraperitoneal injection with vehicle (saline) or NAC (250 mg/kg) 3 times a week for 12 weeks. (A) Urine from ApoE$^{-/-}$ mice were treated with vehicle (n = 5), $NaVO_3$ (n = 10), $NaVO_3$ + 150 mg/kg NAC (n = 5) or $NaVO_3$ + 250 mg/kg NAC (n = 8) were collected and measured by ICP–mass spectrometry. The levels of vanadium were normalized by creatinine (CRE) in urine. (B) Aortas from ApoE$^{-/-}$ mice were treated with vehicle (n = 5), $NaVO_3$ (n = 10), $NaVO_3$ + 150 mg/kg NAC (n = 5) or $NaVO_3$ + 250 mg/kg NAC (n = 8) were examined and photographed under a microscope. White arrows indicate lipid accumulation. (C) Representatively H&E staining of lung tissue from ApoE$^{-/-}$ mice were treated with vehicle (n = 5), $NaVO_3$ (n = 10), $NaVO_3$ + 150 mg/kg NAC (n = 5) or $NaVO_3$ + 250 mg/kg NAC (n = 8). Sections were examined and photographed under a microscope. Black arrows indicate infiltrated immune cells.
